# *Plasmodium vivax* Duffy binding protein: understanding targets of protective immunity

**DOI:** 10.1186/1475-2875-13-S1-O22

**Published:** 2014-09-22

**Authors:** John H Adams, Miriam T George, Francis B Ntumngia, Jesse Schloegel, Samantha J Barnes, Amy M McHenry

**Affiliations:** 1 Global Health Infectious Disease Research Program, Department of Global Health, University of South Florida, Tampa, Florida, USA; 2 Department of Biology & Geology, Southwestern Adventist University, Keene, Texas, USA

## 

*Plasmodium vivax* merozoite invasion of reticulocytes is a rapid process that involves a series of specific, sequential interactions. These interactions are crucial for invasion and continued blood-stage infection, thus representing attractive targets for vaccine development. The Duffy binding protein (DBP) is a vital microneme protein associated with the decisive, irreversible step of junction formation during the later stages of the invasion process. The ligand domain of DBP, known as region II (DBPII), binds the Duffy Antigen Receptor for Chemokines (DARC) by dimerization of two DBP and two DARC molecules forming a heterotetrameric structure. Naturally acquired antibodies to DBPII are prevalent in residents of areas where *vivax* malaria is endemic. These antibodies can inhibit DBPII binding and merozoite invasion of human reticulocytes; however, they tend to be poorly inhibitory, strain-specific, and short-lived. Remarkably, strain-specific serologic activity can be altered by even single amino acid substitutions found in allelic variants of DBPII. While strain-transcending, broadly neutralizing antibodies are less prevalent, there is a significant correlation of their antibody reactivity to certain epitopes and inhibition of DBPII-receptor function. Our data indicate epitopes at or near the dimer interface of DBPII represent the primary targets of naturally occurring protective anti-DBPII antibodies that can inhibit merozoite invasion of host erythrocytes. However, these epitopes also contain most of the prevalent variable residues resulting from DBP allelic diversity. These data are consistent with the hypothesis that DBP variation is an immune evasion mechanism responsible for strain-specific immunity and stable, broadly neutralizing immunity is achieved when antibodies target functionally-conserved epitopes, thereby blocking DBP dimerization and inhibiting invasion. To overcome the natural tendency for eliciting a strain-specific response, synthetic DBPII alleles were evaluated for their ability to broaden efficacy of strain-transcending inhibition against diverse DBPII alleles. In an effort to identify potential conserved, protective epitopes we have prepared and evaluated a panel of anti-DBPII murine monoclonal antibodies. Inhibitory activities of these monoclonal antibodies were characterized with *in vitro* functional assays for DBPII and their respective minimal epitopes were mapped using phage display. These targets of putative strain-transcending inhibition identify epitopes that may be targeted for vaccine-induced anti-DBPII inhibitory antibodies.

